# *Ex-vivo* Kidney Machine Perfusion: Therapeutic Potential

**DOI:** 10.3389/fmed.2021.808719

**Published:** 2021-12-24

**Authors:** Ruta Zulpaite, Povilas Miknevicius, Bettina Leber, Kestutis Strupas, Philipp Stiegler, Peter Schemmer

**Affiliations:** ^1^General, Visceral and Transplant Surgery, Department of Surgery, Medical University of Graz, Graz, Austria; ^2^Faculty of Medicine, Vilnius University, Vilnius, Lithuania

**Keywords:** kidney transplantation, *ex-vivo* machine perfusion, *ex-vivo* therapy, graft preservation, kidney preconditioning, ischemia-reperfusion injury, graft rejection, organ regeneration

## Abstract

Kidney transplantation remains the gold standard treatment for patients suffering from end-stage kidney disease. To meet the constantly growing organ demands grafts donated after circulatory death (DCD) or retrieved from extended criteria donors (ECD) are increasingly utilized. Not surprisingly, usage of those organs is challenging due to their susceptibility to ischemia-reperfusion injury, high immunogenicity, and demanding immune regulation after implantation. Lately, a lot of effort has been put into improvement of kidney preservation strategies. After demonstrating a definite advantage over static cold storage in reduction of delayed graft function rates in randomized-controlled clinical trials, hypothermic machine perfusion has already found its place in clinical practice of kidney transplantation. Nevertheless, an active investigation of perfusion variables, such as temperature (normothermic or subnormothermic), oxygen supply and perfusate composition, is already bringing evidence that *ex-vivo* machine perfusion has a potential not only to maintain kidney viability, but also serve as a platform for organ conditioning, targeted treatment and even improve its quality. Many different therapies, including pharmacological agents, gene therapy, mesenchymal stromal cells, or nanoparticles (NPs), have been successfully delivered directly to the kidney during *ex-vivo* machine perfusion in experimental models, making a big step toward achievement of two main goals in transplant surgery: minimization of graft ischemia-reperfusion injury and reduction of immunogenicity (or even reaching tolerance). In this comprehensive review current state of evidence regarding *ex-vivo* kidney machine perfusion and its capacity in kidney graft treatment is presented. Moreover, challenges in application of these novel techniques in clinical practice are discussed.

## Introduction

Kidney transplantation (Tx) remains the gold-standard treatment for end-stage kidney disease. Due to advanced kidney replacement therapies, improved care, and increased survival of such patients, the number of people on the waiting list for a deceased donor kidney is stably high or growing. In 2020, in the EuroTransplant region, <3,000 deceased donor kidneys and <1,000 living donor kidneys were transplanted while ~11,000 patients were on the active kidney waiting list (1). To meet growing organ demands, utilization of grafts donated after circulatory death (DCD), or retrieved from extended criteria donors (ECD) is unavoidable. The main challenge preventing the expansion of the donor pool is the susceptibility of ECD kidneys for ischemia-reperfusion injury (IRI). Moreover, DCD and ECD grafts are more immunogenic, which leads to difficulties in immune regulation after implantation and a higher risk of acute and chronic rejection (2–4).

The development of organ preservation techniques has the potential to overcome these challenges. Therefore, it has been the main focus of research in the solid organ Tx field for at least 30 years (5, 6). Kidney graft preservation relied on static cold storage (SCS) for a long time (7–9) but increasing donor age and comorbidities urgently require more reliable preservation techniques. Since publication of the largest randomized clinical trial (RCT) comparing hypothermic dynamic kidney machine perfusion (HMP) and SCS (10), HMP took root in routine clinical practice (11–14). HMP improves at least short-term outcomes of all types of kidney grafts, especially of DCD and ECD organs, by achieving the first goal of organ preservation—maintain organ quality until implantation. However, to increase the number of transplantable organs, the main focus now is on the improvement of graft quality, graft conditioning, and repair (6). Extensive research on different *ex-vivo* dynamic preservation temperatures [normothermic (NMP) and subnormothermic (SNMP)], perfusate composition, oxygenation, and perfusion duration revealed that machine perfusion could be used as a tool to treat kidneys prior to implantation by creating nearly-physiological conditions (15). Moreover, machine perfusion seems to be a perfect platform for the various biological and pharmacological agents' delivery directly to the kidney graft. *Ex-vivo* treatment is attractive because it would allow avoiding toxicity caused by systemic recipient treatment and logistic difficulties of the donor therapy (16–18). In addition, some novel promising agents just failed to reach the graft when applied systemically (19–21). This issue could be solved by targeted therapy delivery during *ex-vivo* machine perfusion. Moreover, perfusion parameters and perfusate markers would allow constant evaluation of the kidney state during treatment (22). Multiple experimental and clinical works investigated various *ex-vivo* kidney perfusion therapies for three main goals: (i) to reduce ischemia-reperfusion injury, (ii) reduce the immunogenicity of the kidney graft and (iii) promote healing and regeneration of already injured organ.

In this review, we discuss current evidence of kidney *ex-vivo* perfusion techniques and graft treatment during *ex-vivo* perfusion. Of note, in the overview of *ex-vivo* therapies, we did not include usual perfusate components, such as oxygen, nutrition supplement, heparin, vasodilators, and antibiotics, as this is out of our scope. We aim to outline the newest experimental and clinical studies of kidney *ex-vivo* cell therapy, gene therapy, application of nanotechnologies, delivery of different gasses, biological, and pharmacological agents for kidney treatment and pre-conditioning.

## Overview of Kidney *EX-VIVO* Machine Perfusion Protocols

*Ex-vivo* organ perfusion techniques are usually classified according to the perfusate temperature. Below, we briefly present the concepts and the current protocols of hypothermic and (sub)normothermic kidney machine perfusion. The main differences of perfusion techniques are summarized in Table 1.

**Table 1:** Comparison of different kidney machine perfusion techniques.

	**HMP**	**NMP**	**SNMP**
Temperature	4–10°C	35–39°C	20–32°C
Need of oxygen	+/–	+	+
Perfusate	Without oxygen carrier	With oxygen carrier	With/without oxygen carrier
Cost	Moderate	Expensive	Moderate/expensive
Personnel	Single operator	Team	Single operator/team
Device malfunction	Low risk for graft loss	High risk for graft loss	High risk for graft loss
Graft evaluation	Limited capability	Extensive capability	Moderate capability
Current evidence	Clinically proved	Clinical studies ongoing	Experimental stage
Commercially available devices	LifePort^®^, Waters RM3^®^, kidney assist^®^, waves IGL^®^	Kidney assist^®^	Kidney assist^®^
Therapies/interventions tested	• Cell therapy (MSC) • Gene therapy (siRNA) • Biological therapy (etanercept) • Thrombolytics, fibrinolytics, anticoagulants (tPA, CHC, thrombalexin) • Gases (CORM-401) • Others (metformin, doxycycline, propofol)	• Cell therapy (MSC, MAPC) • Gene therapy (siRNA) • Biological therapy (αCD47ab) • Nanotechnologies (nanoparticles) • Thrombolytics, fibrinolytics, anticoagulants (tPA + plas-minogen) • Gases (CORM-3, Argon, CORM-401) • Cytosorb hemadsorbtion • Others (EPO, cyclic helix B peptide, metformin, SUL-121)	Cell therapy (MSC) Gene therapy (shRNA) Nanotechnologies (NB-LVF4) Gases AP39 (H_2_S donor)

*HMP, hypothermic machine perfusion; NMP, normothermic machine perfusion; SNMP, subnormothermic machine perfusion; MSC, mesenchymal stromal cells; MAPC, multipotent adult progenitor cells; siRNA, small interfering ribonucleic acid; shRNA, short hairpin ribonucleic acid; NB-LVF4, nano-barrier membrane; tPA, tissue plasminogen activator; CHC, corline heparin conjugate; CORM, carbon monoxide releasing molecule; EPO, erythropoietin*.

### Hypothermic Machine Perfusion

During HMP, cold (4–10°C) acellular preservation solution is pumped through the kidney vasculature (5). Several kidney HMP devices are currently commercially available, including LifePort^®^ (Organ Recovery Systems; Itasca, Illinois), Kidney Assist^®^ (OrganAssist; Groningen, Netherlands), Waters RM3^®^ kidney perfusion system (Rochester, Minnesota) and Waves IGL^®^ (Lyon, France). In the current *ex-vivo* perfusion machines, roller or centrifugal pumps are used to generate pressure-controlled pulsatile flow, avoiding perfusion-related graft injury. For kidney HMP, low pressure (25–30 mmHg) is preferable to prevent kidney edema and endothelial damage (5, 23). The perfusion solutions used are qualitatively different from SCS solutions. The only clinically proven fluid for kidney HMP is Kidney Perfusion Solution-1 (KPS-1^®^), which has the same composition as Belzer MPS^®^ UW (Machine Perfusion Solution University of Wisconsin) (14). Unlike the conventional UW solution and other SCS solutions, KPS-1 has an extracellular-like Na+/K+ balance. Moreover, it contains other impermeants (gluconate and mannitol instead of lactobionate and raffinose) and glucose. A variant of commonly used HTK (histidine-tryptophan-ketoglutarate, Custodiol^®^) solution Custodiol-N^®^ supplemented with dextran 40 was used for kidney HMP in several porcine models (24). Moreover, a novel Custodiol-MP^®^ solution has been recently developed exclusively for aerobic machine perfusion (25). Despite promising results of experimental models, further studies are necessary to prove the superiority of Custodiol-N^®^ or Custodiol-MP^®^ over KPS-1^®^ in terms of kidney graft function and transplant outcomes (24, 25).

MP was widely used at the beginning of the solid organ Tx era when the first kidney transplant programs were developed. This method was considered the only safe and reliable way to preserve grafts, especially for long periods of organ storage. These machines were large and difficult to transport (26, 27). Later, Collins et al. presented a method to preserve and transport kidneys on ice in a preservation solution (7). More elaborated preservation solutions granted safe, easy, and much cheaper SCS with satisfactory Tx outcomes decreasing HMP's popularity, inaugurating the era of SCS (7–9). SCS served well as a preservation method of healthy kidney grafts. However, with the growing demand for organs and the aging donor population, the usage of kidneys from ECD lately became unavoidable. The limited applicability of SCS for higher-risk organs encouraged extensive research on alternative and more sophisticated preservation methods. The HMP landmark study in 2009 by Moers et al. brought the machine perfusion back to clinics. This EuroTransplant study, which included 672 kidneys retrieved in 60 European Tx centers, revealed a significantly improved graft survival at 1- and 3-years as well as reduced DGF rates in HMP kidneys compared to SCS (10). Even though this study included mainly donation after brain-death (DBD) grafts, Zhong et al. also found significant benefits in 1- and 3-years graft survival of HMP in the DCD kidneys cohort (28). An independently powered extension of Jochmans et al. initial RCT showed reduced delayed graft function (DGF) rates in DCD kidneys that underwent HMP (29). Similarly, another RCT reported that HMP significantly diminished DGF rates and improved 1-year death-censored graft survival in ECD kidneys in comparison with SCS (30). The superiority of kidney HMP vs. SCS was also confirmed by many systematic reviews and meta-analyses: reduction of DGF rates was observed in both DCD (31, 32) and ECD kidneys (33), and across all donor types (11–14, 34). Similarly, meta-analyses reported improved ECD kidney graft survival at 1-year (33) and among all donor types at 3-years (13). A Cochrane Review, including 16 studies published during the previous 10-years including 2,266 patients, revealed that HMP significantly reduced DGF rates in both DBD and DCD kidney Tx. However, the number needed to treat to prevent one episode of DGF was lower for DCD kidneys (14). Several systematic reviews and meta-analyses demonstrated significant reduction rates in kidney primary non-function (PNF) following HMP compared to SCS (12, 35).Nevertheless, the HMP effect on short- and long-term kidney graft function remains inconclusive (14).

In depth, knowledge about the exact mechanism, how pulsatile hypothermic perfusion improves kidney outcome, is scarce. However, several experimental studies brought some clarity into the field. Similar to SCS, during HMP, the rate of metabolism is reduced to ~10% of physiological temperature. Although HMP does not prevent cold-related depletion of adenosine triphosphate (ATP) and accumulation of metabolic products in the graft (5), the dynamic manner of preservation gives its beneficial effects. Firstly, HMP allows the elimination of debris, toxic metabolites, and free radicals produced during hypothermia (36, 37). Hemodynamic stimulation of the graft vasculature helps prevent endothelial damage, leading to anti-inflammatory effects and may even reduce the graft's immunogenicity. Pulsatile flow generates vascular shear stress, which influences endothelial gene expression and function (5, 38). Moreover, HMP enhances endothelial nitric oxide (NO) synthase (eNOS) phosphorylation, preventing vasospasm and promoting NO-depended vasodilatation at reperfusion. Good microcirculation at the time of implantation vastly increases the chances of immediate graft function (39). On the other hand, even though cellular metabolism during HMP is reduced, it is not entirely ceased. Therefore, the problem of oxygen deficiency remains: a decrease in ATP levels results in inhibition of Na+/K+ pumps and leads to acidosis. Mitochondrial dysfunction and ROS production due to oxygen deficiency promote graft lesions during reperfusion (40). In a porcine DCD model, Kaminski et al. reported a rapid decrease in perfusate oxygen pressure from 150 mmHg at 10 min of perfusion to 6.8 mmHg at 200 min and kidney cortical oxygen pressure dropped from 10.2 to 0 within the first 60 min of perfusion (41). While healthy kidney grafts endure anoxia at low temperature for some time, such conditions may be harmful to ECD or DCD kidneys significantly diminishing the optimal preservation time. Not surprisingly, despite reported superiority of HMP when compared to SCS, RCT and meta-analyses reveal a lack of evidence that HMP improves long-term graft function or long-term survival of DCD kidneys (14, 29). Moreover, even with HMP, the kidney graft outcome depends on cold ischemia time (CIT). Therefore, preservation time cannot be extended (30, 42). HMP itself does not have a conditioning effect and does not improve kidney quality but rather helps maintaining kidney quality as it was at the time of retrieval.

These limitations of HMP led to a growing interest in oxygenated HMP (HMPO_2_). During HMP higher levels of ATP are produced than during SCS, and oxygen is further able to support ATP synthesis (43). Rodent models revealed that oxygenation in HMP reduced nuclear injury, tubular damage, macrophage activation, increased kidney function and even suppressed T-cell response after implantation (37, 44). In pre-clinical porcine models, HMPO_2_ improved early kidney graft function and reduced fibrosis (43, 45–48). The COPE-COMPARE study (49, 50) included 106 paired kidneys retrieved from DCD donors aged ≥50 years and compared HMPO_2_ to standard HMP. It demonstrated that HMPO_2_ is safe and feasible resulting in significantly less severe complications after Tx in the HMPO_2_ group. HMPO_2_-perfused grafts did not show significant improvement in estimated glomerular filtration rate (eGFR) at 12 months post-transplant when comparing functioning kidneys from both groups. However, sensitivity analysis, considering all-cause graft failure, showed significantly improved 1-year kidney graft function in the HMPO_2_ group. Interestingly, HMPO_2_-preserved kidneys had a significant relative risk reduction of acute rejection compared to HMP. No statistically significant difference was seen in terms of DGF and PNF (49). Another clinical trial (COPE-POMP) randomized ECD kidneys to end-HMPO_2_ after SCS vs. SCS alone with graft survival at 12 months post-transplant as a primary endpoint. The minimum HMPO_2_ time was 120 min before implantation. Both groups had comparable 1-year graft survival. Moreover, no significant differences in terms of DGF, PNF, eGFR, and acute rejection between the groups were observed. The overall graft survival rate was high in this study leading to the assumption that the analysis might be underpowered (50). All in all, it seems that oxygen is the key factor when considering HMP not only as a preservation technique but also as an organ conditioning tool. However, additional clinical studies are necessary to confirm this hypothesis.

### Normothermic Machine Perfusion

Despite the benefits of HMP, the detrimental effect of the cold remains, and the kidney graft outcome is still very dependent on CIT. Even after adoption of HMP into clinical practice, the utilization of marginal kidneys, especially DCD, remains limited. The emerging technology of NMP that reduces or even eliminates CIT and harm caused by low temperatures has a great potential in expanding the donor pool (5, 6). During NMP, the organ is perfused with oxygenated blood or other oxygen carrier containing perfusate, nutrients, and medications at body temperature (35–37°C) (5, 6, 15). The preclinical trials by Hosgood and Nicholson found that the optimal arterial pressure for kidney NMP is the lower end of the physiological range for kidney autoregulation [mean arterial pressure (MAP) ~95 mmHg] (51). The main difference between HMP and NMP perfusion solutions is that NMP requires an oxygen carrier. Leukocytes and thrombocytes depleted autologous whole blood has been used for kidney NMP in experimental studies (52–54). Elimination of white cells and platelets allows restoration of organ function without induction of inflammatory reactions and thrombosis, occurring during normal *in-vivo* reperfusion. The remaining plasma contains albumin and globulins, which maintain stable osmotic pressure and electrolytes that regulate pH. However, it can be logistically demanding to harvest autologous blood and prepare perfusate on the spot in clinical situations. Moreover, the remaining fibrinogen may promote micro thrombus formation (15). Therefore, red blood cells (RBC)-based solution is used much more commonly. RBCs efficiently carry oxygen, as well as, their flow reduces shear stress in kidney vessels maintaining normal endothelial function (15) but the increased risk of hemolysis due to RBC contact with artificial surfaces in the perfusion circuit should not be under estimated. Longer-banked RBCs may not be suitable because of higher levels of non-transferrin-bound iron and time-dependent metabolic alterations (15, 55–57). As an alternative, artificial oxygen carriers, including polymerized bovine hemoglobin-based oxygen carriers (HBOC) and pyridoxylated bovine hemoglobin, as well as manufactured, acellular oxygen-carrying media, including Lifor, Aqix RS-I, STEEN solution, have been proposed. The properties of different oxygen carriers are discussed in detail elsewhere (15). In addition, in most of the protocols NMP perfusates contain heparin (if it is blood- or RBC-based perfusate), vasodilators, mannitol, corticosteroids, antibiotics, nutrient preparations with glucose, amino acids and insulin (5, 6, 15).

Such graft preservation in almost physiological conditions enables normal cellular metabolism and recovery of ATP production (58). Some experimental works revealed that 1 h NMP promotes IL-6, IL-8 (59), and heat shock proteins (HSP) expression in kidney grafts (59, 60). Moreover, induction of protective stress responses, down-regulation of cell death, and enhanced proliferation have been observed (61). It has been demonstrated that long periods of NMP can support *de novo* protein synthesis and promote recovery of cytoskeletal integrity in ischemically-damaged kidneys (62). Interestingly, the release of inflammatory cytokines and chemokines into the perfusate and recirculation during NMP has been observed by several investigators (63–65). Transcriptional profiling of NMP perfused kidneys revealed that not only oxidative phosphorylation genes but also many immune and pro-inflammatory pathway genes are significantly up-regulated after 4 h of NMP. Most likely, circulating cytokines promote inflammatory gene expression; therefore, their removal could be beneficial (discussed below) (64, 65). On the other hand, NMP initiates donor-derived T- and B- lymphocytes, natural killer (NK)-cells, macrophages, and granulocytes diapedesis and removal (63).

Current evidence shows, that NMP itself has a therapeutic effect on kidney grafts, though the full mechanism remains to be revealed. In addition, NMP should be an ideal platform for the delivery of various *ex-vivo* therapies as pharmacokinetics and pharmacodynamics of drugs should not be altered by low temperature (5, 6). Another advantage is the possibility of kidney evaluation in physiological conditions before implantation, which could both alleviate decision-making in graft utilization and allow graft assessment during *ex-vivo* therapy (22). Hosgood et al. established a kidney quality score based on renal blood flow (RBF), urine output (UO) during NMP, and macroscopic graft appearance (66). Extensive research is ongoing to find an ideal perfusate or urine biomarker (or a combination of biomarkers) for graft quality and function assessment (22).

So far, most of the experimental and clinical experience has been acquired with short-term pre-implantation kidney NMP. One of the pioneering groups, established the NMP technology using modified pediatric cardiopulmonary bypass equipment and demonstrated its feasibility and superiority over SCS in multiple animal experiments (52, 58, 59). In 2013 they published the first pilot clinical series, in which 18 ECD kidneys underwent 1 h pre-implantation NMP revealing a significantly lower incidence of DGF when compared to the consecutive historical control group (ECD kidneys implanted after SCS) (67). Later they reported successful implantation of a pair of previously considered non-transplantable human kidneys after 1 h NMP at the end of SCS (68). Currently, the same group is conducting the first RCT (ISRCTN15821205), comparing pre-implantation NMP vs. SCS for DCD kidneys. Three hundred thirty-eight patients have been recruited, and results of this trial are expected in 2021 (69). On the other hand, just a few studies have directly compared NMP with HMP or HMPO_2_ so far. Several experimental models demonstrated the superiority of a short period of NMP after HMP (52) or prolonged SCS (58) vs. HMP-only. Contrary to those findings, in the recent study by Vallant et al. porcine kidneys after 4 h of end-ischemic HMP had higher urine output, oxygen consumption, perfusate flow rates, and lower number of apoptotic cells than paired grafts that underwent 4 h of end-ischemic NMP (70). This is in consistence with the results of Darius et al. (46) and Blum et al. (71) who also did not find a beneficial effect of kidney NMP compared to HMP or HMPO_2_ in their experiments. One of the reasons for this inconsistency with previously mentioned results could be different protocols and compositions of preservation solutions. Importantly, another RCT has recently started in the Netherlands, aiming to compare 2 h NMP after HMP vs. HMP-only for DCD or ECD kidneys (NCT04882254).

Currently, Kidney Assist^®^ (OrganAssist; Groningen, Netherlands) is the only commercially available device for kidney NMP. As this machine is not portable, kidney NMP remains a static method and can be used only in combination with SCS or HMP, which are the only commercially available options for organ transportation. Moreover, the optimal duration of NMP is still to be determined. Even though clinically, short-term pre-implantation kidney NMP has been demonstrated as sustainable, the maintenance of NMP for long periods is challenging. One of the main issues is the toxic products unavoidably deriving from the cells' activity that need to be eliminated (70). Moreover, the NMP procedure itself is expensive and requires a lot of human resources; not surprisingly, its cost-effectiveness remains questionable. The risk of the device malfunction, which would lead to organ loss, should also be taken into account. Nevertheless, there is growing evidence demonstrating the benefits of long-term NMP. Recently, a Dutch group registered a new clinical trial PROPER (NCT04693325), aiming to prolong NMP up to 6 h. One of the leading groups in kidney graft preservation at the University of Toronto explored prolonged NMP up to 16 h in porcine experiments. They revealed that long-term normothermic preservation significantly reduces tubular injury and improves kidney function, compared to SCS, HMP or end-ischemia short-term NMP (72–78). The most recent works already demonstrated the safety and feasibility of 24 h kidney NMP, though these grafts were not implanted (79–82). Importantly, Weissenbacher et al. in porcine and non-transplantable human kidneys experiments revealed, that one key factor allowing prolongation of NMP is urine recirculation (80–82). Perfusate homeostasis and stable kidney arterial flow could be achieved only by urine recirculation (80, 82). Moreover, proteomics analysis showed decreased damage-associated molecular patterns, angiotensinogen levels, and enhanced levels of enzymes involved in kidney metabolism, perfused with urine recirculation (81). Pool et al. recently demonstrated that perfusate composition also significantly impact kidney injury and perfusion parameters during prolonged NMP (83). Basile et al. observed stable perfusion parameters and no evidence of damage during 72 h of NMP, revealing that even several days of perfusion could be feasible (79). However, further investigation of principal perfusate components is necessary to establish optimal NMP conditions, allowing long-term NMP. In the studies by Weissenbacher et al. a pre-clinical automatic portable NMP machine was used, confirming that it is only a matter of time until NMP becomes feasible even for organ transportation (80–82).

Although some challenges remain to be overcome, the results of the previously mentioned studies suggest that long periods of NMP may be necessary for re-conditioning of the more severely damaged kidneys. Moreover, the ability to extend *ex-vivo* preservation time could allow applying regenerative techniques and other treatments regimen. It is most likely, that different NMP protocols and perfusate components may be necessary considering different donation situations and kidney quality. Finally, kidney Tx surgery could become an elective daytime procedure.

### Subnormothermic Machine Perfusion

A less investigated but also promising alternative for kidney graft preservation is SNMP. Organ perfusion at 20–32°C aims to avoid cold-induced graft injury but does not increase metabolism to a level requiring oxygen carriers for adequate oxygenation (5). Currently, this technique is still at an experimental stage. One study showed that 1 h end-ischemia kidney perfusion at 37°C preserves tubular and kidney functions better than the same duration perfusion at 32°C, which raised a concern that lower than physiological temperatures may reduce RBC oxygen-carrying capacity (84). However, a study by Hoyer et al. revealed that SNMP is feasible and beneficial even without oxygen carrier: 7 h perfusion of porcine DCD kidneys at 21°C with Custodiol-N, supplemented with dextran 40, resulted in a better-preserved organ structure when compared to oxygenated HMP or SCS. Moreover, a 2-fold increase in creatinine clearance (CrCl) compared to oxygenated HMP, and a 10-fold increase in CrCl, compared to SCS was demonstrated (85). Another group demonstrated that 4 h porcine kidney perfusion with autologous blood at 22°C significantly reduced IRI-related structural injury, hemorrhage and clotting, as well as increased UO and RBF in comparison to kidneys, perfused at 15 and 37°C. Moreover, SNMP diminished the expression of Toll-like receptor signaling molecules [high mobility group box 1 (HMGB1), myeloid differentiation factor 88 (MyD88), nuclear factor kappa-light-chain-enhancer of activated B cells (NF-kB)] and reduced the levels of the kidney injury marker neutrophil gelatinase lipocalin (NGAL) and IL-6 (86). In another study, the same authors showed the feasibility of a hemoglobin-based oxygen carrier (HBOC-201) for 22°C kidney SNMP (87). STEEN solution has also been used for prolonged (24 h) SNMP at 21°C and demonstrated the advantage over 37°C blood-based solutions in terms of vascular resistance during perfusion (88). Brasile et al. used 32°C for kidney perfusion with EMS medium (89).

Moreover, SNMP has been successfully used for delivery of pharmacological agents (90). For example, the cytoprotective property of the H_2_S donor AP29 in University of Wisconsin (UW) solution seemed more pronounced at 21 °C than in hypothermic or normothermic conditions, suggesting that SNMP might be an optimal platform for certain agents delivery (90, 91).

Lastly, a controlled oxygenated rewarming strategy has been proposed as a safer gradual transition from cold to warm before reperfusion, avoiding a sudden heat shock (92–94). According to the protocol of Minor et al. (93) and von Horn and Minor (94) following SCS the kidney is perfused *ex-vivo* starting at 8°C and 30 mmHg and gradually elevating temperature and pressure up to 35°C and 75 mmHg during the first 90 min of 2 h perfusion. Pre-clinical studies demonstrated improved mitochondrial recovery (94) and kidney function following auto-Tx (95) after controlled oxygenated rewarming compared to SCS alone (93). The same group recently demonstrated that 2 h of controlled-oxygenated rewarming after 6 h SCS improved kidney function of ischemically damaged porcine kidneys at a similar level as 8 h NMP (96). One successful Tx of a human ECD kidney after gradual oxygenated rewarming has been reported (93). Despite promising pre-clinical results, further investigation is necessary to prove the applicability of these techniques and establish optimal protocols in terms of timing and perfusate composition.

## Kidney Graft Therapy During *EX-VIVO* Machine Perfusion

Many different pharmacological and biological therapies, nanotechnologies, and hemadsorbtion techniques have been applied for kidney grafts during *ex-vivo* machine perfusion. Experimental and clinical studies investigating kidney *ex-vivo* machine perfusion therapies are summarized in Table 2 and discussed in detail below.

**Table 2:** Summary of kidney *ex-vivo* perfusion therapies.

**References**	**Treatment, dose**	**Model**	**Perfusion parameters**	**Assessment of outcomes**	**Main findings**
**Cell therapy**					
Gregorini et al. (97)	5 × 10^6^ MSC EV derived from 5 × 10^6^ MSC	Rat kidneys; 20 min WI ➔ 4 h HMP MSC *n* = 5; EV *n* = 5; No additives *n* = 5	HMP 4 °C; perfusate: UW (Belzer) solution	Assessment and sampling during HMP	• ^↓^ histological kidney damage score (MSC, EV); • ^↑^ gene pathways for molecular transport, respiratory electron transport and the citric acid cycle (MSC); • ^↑^ Idh2, Ndufs8, Pdhb (MSC, EV); ^↑^ Calb1, Slc16a1, Atp6v0d2 gene expression (EV); • ^↓^ LDH, lactate, MDA, glucose; ^↑^ pyruvate in perfusate (MSC, EV).
Brasile et al. (79)	1 × 10^8^ MSC	Non-transplantable human kidneys; 29.4 ± 7.4 h SCS ➔ 24 h SNMP *N* = 5 kidney pairs	SNMP 32°C; perfusate: exsanguinous metabolic support solution	Assessment and sampling during SNMP	• No MSC migration out of the vasculature into the kidney parenchyma; • ^↑^ ATP; • ^↓^ pro-inflammatory cytokines (eotaxin, G-CSF, IL-6, IP-10, MIP-1a, MIP-1B, RANTES, TNF-α, MCP-3, Flt-3L, GM-CSF, fractalkine, MDC, IL-1B); • ^↑^ growth factors (EGF, FGF-2, TGF-α); • ^↑^ restoration of cytoskeletal integrity (ZO-1 protein location in the cell); • ^↑^ DNA synthesis (PCNA); • ^↑^ mitosis
Pool et al. (98)	1 × 10^5^ human A-MSC 1 × 10^6^ human A-MSC 1 × 10^7^ human A-MSC BM-MSC (fluorescent-labeled) Added after 1 h of NMP	Porcine kidneys; 30 min WI ➔ 3.5–5 h SCS ➔ 7 h NMP. *N* = 3 per group of different concentrations; Labeling and detection: *n* = 10	NMP 37°C; RBC-based perfusate with WME^*^	Assessment and sampling during NMP	• ND in macroscopic changes and haemodynamics; • MSC (when infused ≥ 1 × 10^6^) identified only in the lumen of glomerular capillaries. MSC still intact after tissue engraftment; • Minority of glomeruli positive for fluorescent pre-labeled BM-MSC; • ^↓^ number of MSC in perfusate during NMP (with or without kidney)
Pool et al. (99)	1 × 10^7^ human BM-MSC 1 × 10^7^ human A-MSC Added after 1h of NMP	Porcine kidneys; 20 min WI ➔ 2–3 h oxygenated HMP ➔ 7 h NMP BM-MSC *n* = 5; A-MSC *n* = 5; No additives *n* = 5	NMP: 37°C; RBC-based perfusate^*^	Assessment and sampling during NMP.	• ^↓^ NAG in perfusate (A-MSC vs. control); • ^↓^ LDH (BM-MSC); • ^↓^ NGAL (BM-MSC and A-MSC); • ^↑^ HGF (BM-MSC and A-MSC); • ^↑^ Endothelin-1 (BM-MSC vs. A-MSC); • ^↑^ IL-6 and IL-8 (BM-MSC and A-MSC); • ND in histology scores, arterial flow rate, cumulative diuresis, kidney function
Lohman et al. (100)	1 × 10^6^ porcine A-MSC 1 × 10^6^ human A-MSC	Porcine kidneys; 75 min WI ➔ 14 h oxygenated HMP ➔ 2 h NMP Porcine A-MSC *n* = 7; Human A-MSC *n* = 7; Vehicle group *n* = 7; No additives *n* = 7	NMP^*^	AutoTx after contralateral nephrectomy, follow-up 14 days	• MSC retention in kidney cortex; • No effects on perfusion haemodynamics, no adverse effects after Tx; • ND in plasma crea, GFR, NGAL, kidney damage assessed by histology
Thompson et al. (101)	50 × 10^6^ MAPC Added after 1 h of NMP	Non-transplantable human kidneys MAPC *n* = 5; Control (10 ml bolus of crystaloid) *n* = 5	NMP 36.5 °C; RBC-based perfusate^*^	Assessment and sampling during NMP	^↑^ UO; ^↓^ NGAL, ND in KIM-1 in urine; Restored RBF within the kidney medulla 4 h after MAPC cell infusion (US MicroFlow imaging and CEUS);
					^↓^ IL-1β, ^↑^ IL-10; ^↑^ IDO activity (Kyn:Trp ratio); ND in oxygenation, biochemical parameters, IRR, RBF.
**2. Gene therapy**					
Yuzefovych et al. (102)	Lentiviral vectors encoding shRNA targeting β2-microglobulin (shβ2m) and the class II transactivator (shCIITA) (1.5 × 10^11^ particles)—silencing rat MHC I and MHC II gene expression	Rat kidneys; 10 min SCS ➔ 2 h SNMP (gradual rewarming and increasing flow speed) Treatment group: (shβ2m and shCIITA-encoding vector) *n* = 7; Control group: (vector with non-sense shRNA) *n* = 5	SNMP 31–32 °C; perfusate: WME with 5% BSA^*^	Assessment and sampling during SNMP; AlloTx, follow-up 6 weeks	• ^↓^β2m and CIITA levels; • ND in perfusate LDH levels, histology findings • ^↓^ IL-17 or IFN-γ, IL-12 and MCP-1 levels during entire perfusion time; ^↓^ IL-6 at the beginning, but ^↑^ at 2 h; ^↑^ IL-10, MIP-1a, MIP-2, IP-10, TNF-α, and EGF at 2 h • ND in perfusate RANTES levels
Yang et al. (103)	Naked caspase-3 siRNA. 3 mcg/ml into the renal artery before SCS and 0.15 μg/ml into autologous blood (used for perfusion)	Porcine kidneys; 10 min WI ➔ 24 h SCS ➔ 3 h NMP (“reperfusion”) siRNA *n* = 3; no additives *n* = 3	NMP (“reperfusion”) 38 °C, perfusate: heparinized autologous whole blood^*^	Assessment and sampling during NMP (“reperfusion”)	• ^↓^ caspase-3 precursor and active subunit; ^↓^ active caspase-3 positive cells (40% reduction); • ^↓^ apoptotic cells were; • Marginally ^↓^ MPO positive cells; • Marginally ^↑^ RBF; • ^↑^ oxygen consumption; • Neutralized perfusate pH
Moser et al. (104)	100 μM doxycycline 50 nM MMP-2 inhibiting siRNA	Rat kidneys; 10 min WI ➔ 60 min flushing with 4°C KPS-1 ➔ 22 or 5 h HMP 4–7 rats per group: Treatment group 1 (22 h HMP, doxycycline); Treatment group 2 (22 h HMP, MMP-2 siRNA); Control group 1 (22 h HMP); Control group 2 (5 h HMP)	HMP; 4°C, perfusate: KPS-1	Assessment and sampling during HMP	• ^↓^ MMP-2 in the perfusate (siRNA); • ^↓^ LDH and cytochrome c oxidase in the perfusate (doxycycline); • ^↓^ protein release into the perfusate (doxycycline); • ^↓^ NGAL (doxycycline and MMP-2 siRNA); • Protection of mitochondrial membrane (doxycycline and MMP-2 siRNA)
**3. Biological therapy**					
Diuwe et al. (105)	Etanercept 1.5 mg into the perfusion fluid after the 1st h of HMP	Human kidneys; SCS ➔ HMP. Treatment group *n* = 47; Control group *n* = 47	HMP 4°C; perfusate KPS-1	Tx, follow-up from 12 to 46 months	• ND in patient survival at 12 and 24 months, patient survival with functioning graft at 12 and 24 months; PNF, DGF, immediate function, acute rejection, serum crea levels on day 7 or at months 1, 3, 6, 12, and 24 after Tx; • 2.3-fold ^↑^ risk in the recipient's death, 2.6-fold ^↑^ risk in graft loss, 2.5-fold ^↑^ risk in occurrence of the composite endpoint (occurrence of any event)
Hameed et al. (106)	αCD47ab: 100 μg immediatly after organ flush at the time of retrieval ➔ 200 μg αCD47Ab into the kidney arterial line, immediately prior NMP start	Porcine kidneys. 6 h SCS ➔ 1 h NMP	NMP; 37°C; RBC-based perfusate^*^	Assessment and sampling during NMP	• Addition of αCD47Ab to the cold flush ➔ no binding; • Infusion into the arterial line at the start of NMP ➔ widespread binding along the glomerulus and kidney tubular epithelium (detectable at the end of NMP); • ^↑^ RBF, ^↓^ IRR, ND in oxygen consumption, UO, CrCl, FENa;
					• ND in expression of IL-6, TNF-α, IL-1β, and IL-18; • ^↓^ kidney tubular debris after NMP, ND in other histological parameters; • ^↓^ increase in oxidative stress during NMP, ND in cell death.
**4. Nanotechnologies**					
Brasile et al. (107)	NB-LVF4 100 μg/min 66 μg/g of kidney weight through arterial line	Canine kidneys. Treatment group 1 (only NMP) *n* = 5; Treatment group 2 (NMP and autoTx) *n* = 4; Treatment group 3 (NMP and alloTx) *n* = 4; Control group (NMP and alloTx) *n* = 4	SNMP 32 °C; acellular perfusate^*^	Assessment after NMP, autoTx, and alloTx after both-sides nephrectomy	• Coverage in 90% of the vascular luminal surface (small and large vessels); • No occlusion of vessels, stable perfusion pressures and vascular flow rates; • Normal serum chemistries, electrolyte profiles and hematology, stable crea levels after autoTx; • Delayed onset of rejection (day 30 vs. day 6 in control group)
Tietjen et al. (108)	antiCD31-nanoparticles (NP) 50 mg/ml	Non-transplantable human kidneys; NMP 8.5 h (3 last kidneys after initial experiments) Imaging group *n* = 1; Concentration determination group *n* = 3; Treatment group *n* = 3; Control group (control nanoparticles) *n* = 1	NMP 36 °C; RBC-based perfusate^*^	Assessment during NMP.	• CD31-NP accumulated 5 to 10-fold more than control-NP; • ^↑^ accumulation in glomeruli compared with surrounding interstitial microvasculature
DiRito et al. (109)	ICAM-2-NP with 10 μg/ml plasminogen + 100 μg/kg of kidney weight tPA	Non-transplantable human kidneys SCS➔ 1 h NMP ICAM-2-NP alone; ICAM-2-NP with fibrinolysis; Control-NP-alone; Control-NP with fibrinolysis	NMP 36 °C; RBC-based perfusate^*^	Assessment and sampling during NMP	• In the absence of tPA + plasminogen, ICAM-2-NP and Control-NP accumulated at identical levels at the site of vascular obstructions; • Together with fibrinolytic therapy ^↓^ retention of the non-targeted Control-NP and ~3-fold ^↑^ retention of ICAM2-NPs in the glomeruli and ~20-fold ^↑^ in the microvessels
**5. Thrombolytics, fibrinolytics, anticoagulants**					
Nghiem et al. (110)	tPA 200 mg	Human kidneys with 50% thrombosed glomeruli detected by wedge biopsy. SCS ~ 35.5 h ➔ HMP 7–24 h; *n* = 14	HMP ~4.1°C, perfusate: HTK solution (Custodiol)	Assessment and sampling during HMP (wedge biopsies before and after HMP), Tx	• ^↓^ in thrombosed glomeruli from 50 to 23%; • Perfusion parameters (IRR, flow rate) improved during 7–24 h of HMP
Woodside et al. (111)	tPA 50 mg	Human DCD kidneys; SCS ➔ HMP ≥1 h; tPA *n* = 19; no additives *n* = 19; *24 kidneys implanted to recipients (12 in each group)	HMP 4–6°C; perfusate: IGL Pulsatile Perfusion Solution^*^	Assessment during HMP, implantation	• ND in RBF and IRR during HMP; • Eradicated microthrombi (only 1 tPA group kidney had significant microtrombi after HMP); • ND in DGF, patient survival, death-censored graft survival, crea, GFR, kidney discard rates; • 1 patient in tPA group required reoperation for bleeding from unknown source
DiRito et al. (109)	10 μg/ml plasminogen + 100 μg/kg of kidney weight tPA	Non-transplantable human kidneys; SCS➔ 1 h NMP Plasminogen + tPA *n* = 9; Only tPA *n* = 3; Only plasminogen *n* = 3	NMP 36 °C; RBC-based perfusate^*^	Assessment and sampling during NMP	• completely cleared microvascular obstructions in all tPA +plasminogen kidneys, remaining obstructions in tPA only group, only minor reduction of obstructions in plasminogen goup;
					• ^↑^ vascular resistance in the tPA only, plasminogen only groups at ~30–60 min; stable resistance in tPA + plasminogen group • ^↑^ urine production, ^↓^ NGAL, IL-6, ICAM-1 in tPA+ plasminogen group
Sedigh et al. (112)	Corline heparin conjugate (CHC) 50 mg	Porcine kidneys (DBD model); HMP 20 h Treatment group *n* = 5; Control group (unfractionated heparin) *n* = 5	HMP; 5.0 ± 0.47°C; perfusate: KPS-1	Assessment and sampling during HMP	• CHC successfully binds to the ischemic vessel walls of kidney arteries and tissue; • ND in vascular resistance, histological changes (histological changes were minimal in all samples)
Sedigh et al. (113)	CHC 50 mg	Porcine kidneys (DBD model); HMP 20 h. Treatment group *n* = 6; Control group *n* = 6	HMP; 1–4°C, perfusate KPS-1	Assessment during HMP; *Ex-vivo* normothermic reperfusion for 3 h	• CHC distributed in the glomerular vasculature; • ^↑^ rate of crea decline, ^↑^ UO, ^↓^ serum lactate levels; • ND in HMP parameters, ^↓^ IRR and ^↓^ MAP (needed to reach the same RBF) during NMP; • ^↓^ urinary NGAL levels; • ^↓^ tubular injury, ND in glomerular and vascular morphology • ND in hemostatic dynamics
Hamaoui et al. (114)	Thrombalexin. 2.1/4.2 μM	Porcine kidneys; 15 min WI ➔ 5 h SCS ➔ 4 h stabilization HMP ➔ 1.5 h HMP treatment Treatment group *n* = 19; Control group (unmodified perfusate or anticoagulantly inert “tail only” proteins) *n* = 19 Non-transplantable human kidneys: 78h SCS ➔ 4h HMP; *n* = 2	HMP; perfusate: UW solution	Normothermic hemoreperfusion (NHRP) for 6 h	• Thrombalexin binding and adherence to kidney microvasculature after pre-treatment, continued adherence after 6 h of NHRP; • ^↑^ RBF and perfusion flow index during NHRP; • ND in functional capillary density; 44% larger D capillaries, 50% faster RBC velocity, 3.5 × greater capillary blood flows and perfusion indices; • Marginally ^↓^ lactate and d-dimers during NHRP; • ND in histology, fibrin(ogen) deposition in interstitial tissues and peritubular capillaries; • Human kidneys: ^↑^ RBF and perfusion indices, ^↓^ lactate, d-dimer, fibrinogen levels
**Gases**					
6. Hosgood et al. (115)	Nitric oxid donor sodium nitroprusside (SNP); 25 mg/250 ml 5% glucose administered for 5 min before and during the first hour of NMP at 25 ml/h; Water soluble carbon monoxide releasing molecule (CORM-3); 0.0146 g/60 ml saline infused into the arterial arm for 5 min before and during the first hour of NMP at 50 ml/h	Porcine kidneys; 10 min WI ➔ 16 h SCS ➔ 2 h NMP SNP *n* = 6; CORM-3 *n* = 6; iCORM-3 (inactive) *n* = 6; Control (no intervention) *n* = 6	NMP 39°C	Assessment during NMP; *Ex-vivo* reperfusion with non-leucocyte-depleted autologous blood	During NMP: • ^↑^ RBF, ^↑^ IRR fall (SNP and CORM-3 vs. controls); During reperfusion: • ^↑^ RBF (CORM-3 vs. control), ^↓^ IRR (SNP vs. controls); • ^↑^ crea fall (SNP and CORM-3 vs. iCORM-3); • ^↓^ UO (SNP and iCORM-3 vs. CORM-3 and control); • ^↓^ oxygen consumption at 3 h (SNP and control vs. CORM-3); • ^↑^ tubular dilatation and vacuolation, number of condensed tubular nuclei (SNP vs. CORM-3)
Smith et al. (116)	Argon (70% Ar, 25% O_2_, 5% CO_2_)	Porcine kidneys; 15 min WI ➔ 17 h SCS ➔ 1 h NMP ➔ 30 min SCS Treatment group *n* = 6; Nitrogen control (70% N2, 25% O_2_, 5% CO_2_) *n* = 6; Oxygen control (95% O_2_, 5% CO_2_,) *n* = 6	NMP 38°C; perfusate: leukocyte depleted blood	Assessment and sampling during NMP; 3 h warm *ex-vivo* reperfusion with autologous blood	• Numerically ^↑^ RBF during NMP in argon and nitrogen groups; • ND in oxygen consumption, CrCl, total UO; • ND in urinary cytokines (IL-6, IL-8, TNF-α), urinary HIF-1α; • ND in HIF-1α tubular cytoplasmic and nuclear staining; • ND in morphology
Bhattacharjee et al. (117)	CORM-401 200 μM (in Plasmalyte solution) after HMP delivered through pulsatile action at 37 °C, over the period of 20 min	Porcine kidneys; 60 min WI ➔ 4 h HMP ➔ 10 h NMP Treatment group: *n* = 5; Control group (iCORM-401): *n* = 5	HMP 4 °C; perfusate: UW solution; NMP 37 °C; perfusate: isogeneic oxygenated blood	Assessment and sampling during NMP (reperfusion)	• ^↓^ ATN score, intrarenal hemorrhage, apoptosis; • ^↓^ KIM-1 and NGAL in urine; • ^↑^ RBF, total UO; • ^↓^ urinary protein, ^↑^ CrCl; • ^↓^ gene expression of TLR2 and 6, MyD88, NF-κB and HMGB1
Juriasingani et al. (90)	20 nM AP39 (H_2_S donor)	Porcine kidneys; 30 min WI ➔ 4 h SNMP Treatment group (SNTAP) *n* = 7; Control group 1 (SNMP without treatment) *n* = 6; Control group 2 (SCS) *n* = 7	SNMP 31°C; perfusate: UW solution and non-stressed autologous blood	Assessment during SNMP; 4 h *ex-vivo* reperfusion at 37°C with stressed autologous blood	• ^↑^ UO during preservation and reperfusion; • ND in ATN score; ^↓^ apoptosis; • 214 genes differentially expressed in SNTAP vs. SCS groups (^↓^ pro-apoptotic (BCL10), heat shock response (HSPD1 and HSPA1A) genes, regulators of those pathways (BAG3, DDIT3); ^↑^ proliferation (MAPK7) and oxidative stress response (NRROS) genes; • 614 genes differentially expressed in the SNTAP group vs. SNMP group, including ^↓^ genes associated with the HIF-1α-mediated hypoxia response pathway (EGR1, PCK1, PDK3, RGCC); ^↓^ proinflammatory (IL6, HMGB2) and pro-cell death (HOXD8, HOXD10) genes; ^↑^ genes mediating TGF-β pathway (SMAD3 and NRROS) and HIF-1α degradation (AJUBA); ^↑^ proliferation (MAPK7) and oxidative stress response (NRROS) genes
**7. Other pharmacological agents**					
Yang et al. (118)	Erythropoietin (EPO) 5,000 U/l	Porcine kidneys; 10 min WI ➔ 16 h SCS ➔ 2 h NMP. Treatment group *n* = 3; Control group (no additives) *n* = 3	NMP 38°C; perfusate heparinized leucocyte-depleted autologous blood^*^	Assessment and sampling during NMP	• ^↓^ apoptotic cells in tubular areas; • ^↓^ macrophages (ML1P+); ^↑^ neutrophils (MPO+) in tubular lumens, but ^↓^ in interstitial areas; • ^↑^ apoptosis of neutrophils; • ^↑^ caspase-3 activity, expression of 17 kD active caspase-3; • ^↓^ IL-1β active protein, ^↑^ its precursor; • ^↓^ tubular dilation and cytoplasmic vacuolisation in tubular epithelium; • ^↑^ UOt; • ^↓^ WBC in hemoperfusate, ND in Hgb level; • ND in RBF, oxygen consumption
Yang et al. (119)	Cyclic helix B peptide (CHBP) 10.56 nmol/l in flushing solution and perfusate	Porcine kidneys; 20 min WI ➔ 18 h SCS ➔ 3 h NMP (reperfusion) Treatment group *n* = 5; Control group (no additives) *n* = 5	NMP 37°C, perfusate: whole heparinized autologous blood^*^	Assessment and sampling during NMP (reperfusion)	• ^↑^ RBF, oxygen consumption, UO at 2 h; • ^↓^ serum potassium; • ND in serum crea level, CrCl, serum pH, ALT, AST, LDH;
					• ^↓^ tubular epithelial vacuolation, tubular dilatation, interstitial expansion; • ^↓^ apoptotic cells in tubular areas, but ^↑^ in interstitium and tubular lumens; • ^↓^ MPO positive cells, expression of caspase-3; • ND in HSP70 expression
Huijink et al. (120)	Rats: Metformin 300 mg/kg for donor 12 and 2 h before kidney retrieval; 30 or 300 mg/l added to NMP perfusate Pigs: Metformin 4 mg/l to HMP perfusate and 20 mg/ml increasing dose to NMP perfusate	Rat kidneys; 15 min WI ➔ 24 h SCS ➔ 90 min NMP Group 1: no metformin *n* = 5; Group 2: metformin for donor *n* = 5; Group 3: 30 mg/l metformin in NMP; Group 4: metformin for donor and 30 mg/l in NMP; Group 5: 300 mg/l metformin in NMP; Group 6: metformin for donor and 300 mg/l in NMP; Porcine kidneys; 30 min WI ➔ 3 h HMP ➔ 4 h NMP. Group 1: no metformin *n* = 6; Group 2: metformin in HMP and NMP *n* = 7	Rats: NMP 37°C, perfusate: WME; Pigs: HMP°C, perfusate: UW Machine Perfusion Solution; NMP 37°C, perfusate: leucocyte depleted, autologous, blood	Assessment and sampling during perfusion	• ND in perfusion parameters, CrCl (rats, pigs); • ^↑^ total UO in metformin-preconditioned rat kidneys; ^↓^ total UO in metformin-perfused rat and porcine kidneys; • ^↓^ protein excretion in metformin-preconditioned rat kidneys vs. control; ND between other groups; • ^↓^ LDH in metformin-preconditioned and/or metformin-perfused (30 mg/l) rat kidneys. ND in porcine kidneys; • ^↓^ tubular necrosis, proximal tubular cell vacuolation (rats); ND in porcine kidneys histology • ^↓^ EDN-1 (rats, 300 mg/l); ^↓^ eNOS (rats, 30 mg/l), ^↓^ VCAM-1 (rats, 30 mg/l), ^↓^ IL-6 (rats, 30 mg/l) • ^↑^HSP70 (pigs)
Moser et al. (121)	100 μM doxycycline	Rat kidneys; 10 min WI ➔ 60 min flushing with 4°C KPS-1 ➔ 22 h or 5 h HMP Treatment group (22 h HMP) *n* = 4; Control group 1 (22 h HMP) *n* = 4; Control group 2 (5 h HMP) *n* = 4	HMP 4 °C, perfusate: 20 ml KPS-1	Assessment and sampling during HMP	• ^↓^ LDH, NGAL and total protein increase at 22 h HMP; • ^↓^ cells separation and extracellular space enlarging, caused by HMP; • ^↓^ mitochondria damage and formation of dense bodies; • ^↑^ levels of triosephosphate isomerase (TPI), phosphoglycerate mutase (PGM), dihydropteridine reductase-2, pyridine nucleotide-disulfide oxidoreductase, phosphotriesterase- related protein, aminoacylase-1A,N(G),N(G) dimethylarginine dimethylaminohydrolase, and phosphoglycerate kinase 1
Nakladal et al. (122).	50 μM SUL-121, SUL-150 [*(R)*-enantiomer of SUL-121] and SUL-151 [*(S)*-enantiomer of SUL-121]	Porcine kidneys; 24 h SCS ➔ NMP	NMP 37°C; perfusate: oxygenated medium (RPMI 1640)	Assessment during NMP	• ^↓^ intrarenal pressure (profound effect with SUL-121 and SUL-150, only a minor effect with SUL-151); • 35% ^↑^ in RBF from baseline with fixed pressure (80 mmHg) (SUL-150)
Snoeijs et al. (123)	Water-soluble (cyclodextrin-complexed) propofol:	Porcine kidneys; 45 min WI ➔ 22 h HMP; Treatment *n* = 6; Control *n* = 6	HMP 4 °C; perfusate: UW solution	AutoTx, 10 days follow-up	• ^↓^ increase in MDA conc. after reperfusion;
	40 μmol/100 mg kidney weight during flush-out and 32 μmol/100 mg kidney weight at 1 h after the start and 1 h before the end of HMP				• ^↓^ increase in IRR at the beginning of reperfusion; • ^↓^ crea concentration after 10 days; • ND in histological changes; • ND the extent of neutrophil infiltration and peritubular capillary inflammation; • No adverse events
**8. Hemadsorbtion**					
Hosgood et al. (64)	Cytosorb hemadsorption	Porcine kidneys; 22 h SCS ➔ 6 h NMP; Treatment group *n* = 5; Control group *n* = 5	NMP 37.4 °C; whole blood-based perfusate^*^	Assessment and sampling during NMP	• ^↓^ RBF at 30 min; ^↑^ mean RBF, oxygen consumption at 1 h (sign.) and 3 h (numerically) but not at 6 h • ND in UO, CrCl, FENa; • ^↓^ urinary NGAL • ^↑^ IL-1β, IL-1α, IL-1RA, TNFα, IL-10 in the control group throughout perfusion, whereas levels remained low in the treatment group; • ^↓^ IL-6 at 3 and 6 h. Numerically ^↓^ IL-8 at 6 h; ^↓^ CRP at 1, 3, and 6 h • ^↓^ thromboxane B2, PGE, prostacyclin
Ferdinand et al. (65)	Cytosorb hemadsorption	Non-transplantable human kidneys; 4 h NMP Treatment group *n* = 5; Control group *n* = 5	NMP 37.4°C; whole blood-based perfusate^*^	Assessment and sampling during NMP. Indirect comparison with human kidneys transplanted after 1 h of NMP	• ND in RBF, UO, oxygen consumption, acid-base homeostasis during NMP; • 46 genes sign. ^↑^ and 181 ^↓^ in the treatment group after 4 h of NMP; • ^↓^ transcriptional response included NLRP3 inflammasome activation-associated genes and neutrophil recruiting chemokines; • ^↓^ “TNFα signaling *via* NFκB” pathway, ^↑^ OXPHOS and fatty acid metabolism pathways (this gene signature was associated with lower incidence of prolonged DGF in transplanted kidneys)

↑ *increase;*

↓ *decrease;*

*ND, no difference; MSC, mesenchymal stromal cells (A-adipose-derived, BM-bone marrow-derived); EV, extracellular vesicles; WI, warm ischemia; HMP, hypothermic machine perfusion; SNMP, subnormothermic perfusion; NMP, normothermic perfusion; UW, University of Wisconsin; ATP, adenosine triphosphate; crea, creatinine; G-CSF, granulocyte colony stimulating factor; IL, interleukin; IP-10, interferon gamma-induced protein-10; MIP, macrophage inflammatory protein; RANTES, Regulated upon Activation, Normal T Cell Expressed and Presumably Secreted; TNF, tumor necrosis factor; MCP, monocyte chemotactic protein; Flt-3L, Fms Related Receptor Tyrosine Kinase 3 Ligand; GM-CSF, granulocyte-macrophage colony-stimulating factor; MDC, macrophage-derived chemokine; EGF, epidermal growth factor; FGF, fibroblast growth factor; TGF, tumor growth factor; ZO, zonula occludens; DNA, deoxyribonucleic acid; PCNA, proliferating cell nuclear antigen; WME, William's E Medium; RBC, red blood cell; NAG, N-acetyl-β-d-glycosaminidase; LDH, lactate dehydrogenase; NGAL, neutrophil gelatinase-associated lipocalin; HGF, hepatocyte growth factor; Tx, transplantation; GFR, glomerular filtration rate; MAPC, multipotent adult progenitor cells; KIM, kidney injury molecule; RBF, renal blood flow; US, ultrasound; CEUS, contrast-enhanced ultrasound; IDO, indoleamine 2.3-dioxygenase; kyn, kynurenine; trp, tryptophan; (sh)RNA, (short hairpin) ribonuclear acid; MHC, major histocompatibility complex; SCS, static cold storage; BSA, bovine serum albumin; IFN, interferon; siRNA, small interfering ribonucleic acid; MPO, myeloperoxidase; MMP, matrix metalloproteinase; KPS, kidney perfusion solution; PNF, primary non-function; DGF, delayed graft function; CD, cluster of differentiation; ab, antibody; IRR, intrarenal resistance; UO, urine output; CrCl, creatinine clearance; FENa, fractional excretion of sodium; NB-LVF4, nano-barrier membrane; ICAM, intracellular adhesion molecule; tPA, tissue plasminogen activator; DCD, donated after circulatory death; DBD, donated after brain death; MAP, mean arterial pressure; HTK, histidine tryptophan ketoglutarate; AST, aspartate aminotransferase; HIF, hypoxia inducible factor; ATN, acute tubular necrosis; TLR, Toll-like receptor; MyD88, myeloid differentiation factor 88; NF-κB, nuclear factor kappa-light-chain-enhancer of activated B cells; HMGB, high mobility group; WBC, white blood cells; Hgb, hemoglobine; ALT, alanine aminotransferase; HSP, heat shock protein; EDN, eosinophil-derived neurotoxin; eNOS, endothelial nitric oxide synthase; VCAM, vascular cell adhesion molecule; CRP, C reactive protein; PGE, prostaglandin E; OXPHPOS, oxidative phosphorylation*.

* *Perfusate includes more components, such as heparin, nutrition agents, antibiotics, vasodilators, etc*.

### Cell Therapy

One of the emerging strategies in *ex-vivo* machine perfusion therapeutics is cell therapy. Mesenchymal stromal cells (MSC) are multipotent cells mainly derived from bone marrow and adipose tissue, but also present in the umbilical cord, placenta, peripheral blood, and other tissues. Due to their stem-cell-like properties, after the engraftment into organs, they can differentiate into various functional cells and interfere with detrimental pathophysiological processes (124). International Society for Cellular Therapy established criteria defining MSC, including plastic-adherence when maintained in standard culture conditions, expression of cluster of differentiation (CD)73, CD90, and CD105 surface markers, lack of expression of endothelial and blood cell markers CD45, CD34, CD14 or CD11b, CD79α or CD19, and human leukocyte antigen (HLA)-DR, and capacity to differentiate into osteoblasts, chondroblasts, and adipocytes *in vitro* (125). Low immunogenicity (the lack of HLA class II expression, low HLA class I and costimulatory molecules expression), immunomodulatory and regenerative properties, as well as unelaborate growth *in vitro* (126) make MSC an attractive therapeutic agent in the field of solid organ Tx (124, 127). Like stem cells, they are able to differentiate into specified cells or deliver organelles to injured cells and consequently restore the function of the damaged organ (128). However, the secretion of growth factors, cytokines, and extracellular vesicles (EV) containing lipids, proteins, mRNAs, miRNAs, non-coding RNAs, and sometimes genomic DNA as well as the modulation of the graft's microenvironment are probably even more crucial mechanisms of action (129–132). Specifically, MSCs may not directly replace damaged kidney epithelial cells, but rather MSCs secretome may promote kidney regeneration from residual mature kidney cells, which become capable, dedifferentiate, and replicate (133). Nevertheless, it has been revealed that not only paracrine activity but also direct cell-to-cell interactions between MSC and host cells play a crucial role in acquiring regenerative, anti-inflammatory, and pro-tolerogenic effects in the target organ (127).

Several clinical kidney Tx trials already showed that systemic recipient treatment with autologous or allogenic MSC modulates the immune response and allows reducing doses of immunosuppressive drugs (134–136).Those studies focused on systemic immune modulation but not on the reparative or regenerative capacities of MSC therapy. Despite promising results, including safety and feasibility, it has become clear that intravenous infusion of MSC might not be ideal for several reasons. Firstly, it has been observed that the majority of intravenously delivered MSC are trapped in the microcapillary system of the lungs and liver hence not reaching the target organ (19–21). Another major problem is the short life span of intravenously infused MSC, meaning that multiple infusions may be necessary (19, 21). On the other hand, intra-arterial delivery of MSC directly to the kidney graft is feasible, efficient, prolongs MSC survival and cell-to-cell contact *in situ*, as well as, off-target migration of infused cells is minimal (137, 138), supporting the idea that cell therapy could be successfully applied for organ preconditioning and repair *ex-vivo* prior to implantation.

Five experimental studies in which MSCs were delivered into the kidney *via ex-vivo* machine perfusion have been published (79, 97–100). Gregorini et al. perfused rat kidneys, harvested after 20 min of warm ischemia, for 4 h with hypothermic UW solution containing MSC or extracellular vesicles derived from MSC. Both treatments significantly reduced histological lesions such as bleb formation, tubular necrosis, tubular lumen obstruction, and overall diminished global kidney damage score after HMP. Moreover, gene sets and individual genes responsible for molecular transport, citric acid cycle, respiratory electron transport, and antioxidant activity were significantly up-regulated. Correspondingly, perfusate biochemical analysis showed significantly lower levels of ischemia markers lactate and lactate dehydrogenase (LDH), oxidative stress marker malondialdehyde (MDA), increased pyruvate, and decreased glucose levels, demonstrating more active kidney metabolism during HMP. Importantly the positive effect was even more considerable in the EV group, compared to MSC-perfused kidneys. It suggests that prompt and direct delivery of free soluble EV mediators could be beneficial during short-term *ex-vivo* perfusion (97). Brasile et al. used 24 h NMP (so-called exsanguinous metabolic support) to deliver MSC to non-transplantable human kidneys focussing on the regenerative potentiality of this therapy. Infusion of 10^8^ MSC (higher doses led to higher MAP, lower vascular flow, and diminished oxygen consumption) resulted in significantly increased ATP concentration in both kidney cortex and medulla, reduced synthesis of pro-inflammatory cytokines, and increased synthesis of growth factors. Moreover, up-regulated DNA synthesis and increased incidence of mitosis associated with MSC therapy were observed. Interestingly, 24 h NMP alone normalized the cytoskeletal integrity of injured kidneys, and MSC therapy further improved this restoration by 4.81%. During 24 h of perfusion, MSC remained in the vascular compartment of the kidney and did not migrate to parenchyma, as proved by histological evaluation and perfusate investigation (79). Similarly, in the study by Pool et al. human MSC stayed in glomerular capillaries of porcine DCD kidneys and remained undamaged throughout 7 h of NMP. Nevertheless, MSC accumulated only in the minority of glomeruli rather than distributed uniformly, even in well-perfused kidneys. Moreover, in this study, a gradual decrease of MSC counts in the perfusate was observed in experiments with kidney as well as without kidney connected to the perfusion machine. That suggests that MSC might be susceptible to perfusion conditions, such as flow and pressure, suggesting to be a limitation of long-term machine perfusion (98). In contrast, Brasile et al. could recover more than 95% of initially infused MSC from the perfusate at the end of 24 h perfusion (79). However, it should be taken into account that Pool et al. used human-derived MSC in porcine kidneys, so there could be a possibility of a xeno-effect, even for leukocyte-depleted perfusate lacking some immune components (98). In a later study the same group demonstrated that porcine DCD kidneys, 7 h perfused with MSC in 37°C, presented with lower levels of kidney injury markers (NGAL and LDH) and increased levels of hepatocyte growth factor (HGF), as well as immunomodulatory cytokines IL-6 and IL-8 in the perfusate. No differences in kidney function or diuresis were observed, which could also be explained by the relatively short perfusion time. Importantly, no apparent differences between adipose and bone marrow-derived MSC were found (99), even though several studies suggested that MSC of different sources may differ in activity and treatment effects (139, 140). The main drawback of the before-mentioned experiments is the lack of data from kidney assessment after reperfusion *in vivo*. Recently, Lohman et al. published their results of porcine DCD kidney auto-Tx after 14 h oxygenated HMP and subsequent 4 h NMP with human or porcine adipose-derived MSC. After 14 days of follow-up, no beneficial effects on kidney function or kidney injury markers were observed. Nevertheless, the treatment neither negatively affected perfusion parameters nor caused adverse events after auto-Tx, thus encouraging further machine perfusion studies with subsequent Tx *in vivo* to investigate MSC impact on ischemia-reperfusion injury, organ regeneration capacity, and immune modulation (100).

Another type of cells, potentially useful for kidney treatment during *ex-vivo* machine perfusion, are multipotent adult progenitor cells (MAPC). Genetically MAPCs are similar to MSCs, reside in the bone marrow, and even have comparable function and mechanism of action. Particular growth and expansion characteristics lead to phenotypically different features of those two cells' populations (141). MAPC immunomodulatory capacity has already been demonstrated in a couple of Tx studies. Treatment with allogenic MAPC in a rat heterotopic heart transplant model allowed to withdraw pharmacological immunosuppressive therapy and successfully achieve long-term survival (142). MAPCs have also been successfully used in a human liver Tx case, which resulted in a pro-tolerogenic profile of the recipient‘s leucocytes and reduced immunogenicity (143). Thompson et al., in their very recent study, used *ex-vivo* NMP to deliver MAPC to non-transplantable human kidneys. MAPC treatment resulted in higher UO, lower NGAL concentration in perfusate, but not other kidney injury biomarkers kidney injury molecule-1 (KIM-1) and flavine mononucleotide (FMN). MAPC was also associated with the changes in cytokine profile—decreased IL-1β and increased IL-10 levels, as well as up-regulated indoleamine-2,3-dioxygenase activity, which is known for its role in pro-tolerant mechanisms and suppression of inflammatory processes (101).

Although the results of machine perfusion cell-therapy studies are promising, several questions remain to be answered. The fate of *ex-vivo* delivered MSC or MAPC is still not determined. It is known that their lifespan in the target organ is limited; however, there is evidence that once immunomodulatory processes have been promoted, these beneficial effects are maintained even after inactivation or death of MSC (144, 145). Therefore, it is necessary to investigate if supportive cell therapy would be beneficial after implantation and at which time points it would be the most efficient. Additionally, it is crucial to thoroughly check into possible immunogenicity of allogenic MSC and MAPC in the machine perfusion setting, as extraction and preparation of autologous cells in case of Tx is usually logistically demanding (146). The perfect duration and conditions of machine perfusion also need to be determined. As discussed previously, several hours of *ex-vivo* cell therapy could be sufficient to promote immunomodulatory and anti-inflammatory processes; however, apparently a much longer time is needed for kidney regeneration and repair (79, 99, 101). Recent study revealed that machine perfusion conditions also affect MSC viability, metabolism, and function, which may not allow reaching the maximum effect of cell therapy (147). Therefore, further work on machine perfusion prolongation, *ex-vivo* organ viability maintenance, and optimal conditions for therapeutic cells is needed.

### Gene Therapy

*Ex-vivo* machine perfusion is a promising platform for organ-specific gene therapy or even genetic engineering. One of the emerging approaches is posttranscriptional gene silencing with small interfering double-stranded RNA (siRNA), which induces degradation of homologous mRNA transcripts and blocks the desired gene expression (148, 149). They can be transported by viral vectors or just injected as a synthetic “naked” form. The main advantage of siRNA, as a tool for gene therapy, is its simple delivery—they are small and do not need to cross the nucleus membrane to become active. siRNA, silencing the expression of RelB, Caspase 3, IKKb, Fas or complement genes, delivery by simple intravenous, or hydrodynamic injection or *via* renal artery, has been already successfully applied in multiple rodent ischemia-reperfusion models (148–152).

However, even though gene therapy techniques, including siRNA, have been developing for the past 20 years, they remain in the experimental stage, not proceeding to clinics. The main problem is low efficiency and insufficient organ specificity of non-viral or viral gene therapy *in vivo* (153). Short lasting effects due to rapid degradation and excretion of the agent, delivered *via* the systemic route, is challenging too. Therefore, *ex-vivo* machine perfusion of the graft could help overcome those drawbacks by specific and efficient application of the gene therapy hence avoiding off-target effects (154).

Yang et al. published the first *ex-vivo* kidney perfusion study with siRNA—naked synthetic caspase-3 siRNA was infused directly into the renal arteries of ischemic porcine kidneys prior to 24 h of SCS followed by addition to autologous blood perfusate used for 3 h NMP. As expected, this treatment significantly reduced caspase-3 precursor and active subunit expression in perfused kidneys. Moreover, treated grafts had 40% lower number of apoptotic cells, shown by histology and immunohistochemistry (IHC). The caspase-3 siRNA group also demonstrated marginally improved RBF, significantly increased oxygen consumption, and improved perfusate pH regulation at 3 h of perfusion. However, no difference in CrCl and UO was observed (103). In another study, siRNA inhibiting matrix metalloproteinase-2 (MMP-2) gene expression was added to HMP perfusate. Ischemic rat kidneys were perfused for 22 h, which diminished MMP-2 and NGAL levels in perfusate to a level similar to that observed at 5 h of perfusion without treatment. Moreover, protection of mitochondrial membranes was observed (104). It suggests that gene therapy could help prolong organ preservation time until implantation and protect from additional injury. Recently, Yuzefovych et al. (102) adopted a similar technique to reduce the immunogenicity of the rat kidney allografts by silencing rat MHC I and MHC II expression. Rat kidneys underwent 2 h of SNMP with short hairpin RNA (shRNA), designed to target rat β2-microglobulin (β2m) and rat class II transactivator genes (CIITA) and carried by a lentiviral vector. Kidneys were implanted, and recipients were followed up for 6 weeks. As a result, transcript levels of β2m and CIITA were reduced by 71 and 70%, respectively. As the vector contained the sequence for nanoluciferase, the bioluminescence activity in plasma and urine was detectable during the whole 6 weeks after Tx, confirming a stable transferred gene expression. Moreover, a cytokine shift toward a pro-tolerogenic milieu was detected in perfusate during perfusion [increased secretion of IL-10, macrophage inflammatory protein (MIP)-1a, MIP-2, interferon γ-induced protein (IP)-10, epidermal growth factor (EGF), and decreased IL-12, IL-17, monocyte chemoattractant protein (MCP-1), interferon γ (IFN-γ)]. Importantly, no vector-related damage of the kidney allograft, as confirmed by LDH levels and histological investigation became evident. Even more, as no off-target distribution of the vector was observed, this diminishes the burden of risks associated with a lentiviral vector, such as tumorigenesis and other systemic side effects (102, 155).

Undoubtedly, these results are a step forward toward the goal to make a kidney graft immunologically invisible and reduce or even eliminate the need for systemic immune suppression for the recipient. Nevertheless, the long-term immunological state of the recipient, the incidence of acute and chronic rejection, the need for pharmacological immune suppression, graft function, and other clinical questions were not investigated in this study and remain to be answered.

### Biological Therapy

The idea to use *ex-vivo* kidney machine perfusion to modulate the biological response of the graft before Tx by administering biological agents into perfusion solution has recently emerged. Inhibition of pro-inflammatory molecules, or their precursors' secretion and action by using specific monoclonal antibodies, blocking target sites, or even changing gene expression at the organ level before implantation gives a rationale that IRI, immunogenicity, and, therefore, need for systemic therapies could be reduced (105, 106). In a clinical study by Diuwe et al. (105) the tumor necrosis factor (TNF)-α inhibitor Etanercept was added to HMP of human kidneys, which were subsequently implanted. Although no negative impact on perfusion parameters was observed, no difference in patient survival at 12 and 24 months, PNF, DGF, an immediate graft function, acute rejection, or serum creatinine levels could be found. Unexpectedly, the proportional hazard Cox model showed that etanercept caused a 2.3-fold increase in the risk of recipient's death and a 2.6-fold increased risk of graft loss. It should be taken into account that all recipients still received a standard immunosuppression regimen of three drugs: corticosteroid, tacrolimus, and mycophenolic acid ester or sodium salt, therefore this study was not able to determine if the need or at least dosage of systemic immune suppression could be reduced by local biological therapy during machine perfusion. Moreover, it is not clear if, in the hypothermic environment, the highest effectiveness of the TNF-α inhibitor could be reached. It is more likely that in higher temperatures, the bioavailability and activity of the drug may be different. Therefore, adoption of (S)NMP into clinical practice would allow more efficient testing of drugs and determining optimal conditions for *ex-*vivo application of biological agents (105). Recently, an experimental study using αCD47Ab, an inhibitor of thrombospondin mediated IRI signaling, was conducted. One dose of αCD47Ab was infused *via* the renal artery immediately following cold perfusion of porcine kidneys at the time of retrieval, while another dose was added to NMP *via* the arterial line. Interestingly, the addition of αCD47Ab into the cold solution did not result in antibody binding to the kidney structures, whereas after 1 h of NMP containing the agent, αCD47Ab was detectable widely spread along the glomerulus and kidney tubular epithelium. Moreover, increased RBF and lower intrarenal resistance (IRR) during NMP could be observed in treated kidneys. However, oxygen consumption, UO, CrCl, and fractional sodium excretion (FENa) did not differ from control organs. Histologically, a significant increase in tubular dilatation and vacuolation in 1 h of NMP was detected in all kidneys whereas αCD47Ab-treated organs had reduced kidney tubular debris. NMP-induced oxidative stress was reduced in treated kidneys but the extent of cell death remained similar in comparison to control kidneys. Although no reperfusion was performed and the beneficial effects of this treatment on IRI remain questionable, the finding of successful delivery and binding of the antibody to graft structures during only 1 h of NMP encourages further investigation of machine perfusion as a platform for organ-targeted biological therapy (106).

### Nanotechnologies

*Ex-vivo* machine perfusion is an attractive platform to apply novel nanotechnology in the solid organ Tx field. The main limitation of its use *in vivo* is some difficulties with systemic administration. NPs are not able to escape from the bloodstream and reach extravascular targets (156, 157). Moreover, they tend to be trapped in liver and spleen phagocytes (158) or be absorbed by serum proteins and form “protein corona “which also disturbs specific targeting (159). Therefore, delivery of NPs directly to the graft in leukocyte-depleted, serum-free perfusate is promising.

So far, endothelial cells of kidney graft vasculature have been chosen as the main target for nanomedicine. Firstly, because the endothelium is the primary point, where ischemia-reperfusion or immune response-caused graft injury starts and secondly because it is directly accessible to the perfusate (107, 108). Brasile et al. used SNMP of 32 °C as a platform to deliver a receptor-mediated bioengineered nano-barrier membrane (NB-LVF4), made of laminin, vitrogen, fibronectin, and type IV collagen, to canine kidney grafts, to “immunocloak” the vasculature and reduce the antigenicity of the endothelium. The main idea was to provide a physical nano-barrier between recipient's immune cells and graft endothelium without interfering with the transport of nutrients and oxygen and thus making the vascular surface non-immunogenic and non-thrombogenic. Three hours of perfusion with the agent allowed a coverage of 90% of small and large kidney vessels without any occlusion or disturbances in perfusion parameters. Autotransplanted kidneys revealed a good function, proving that NB-LVF4 local treatment *ex-vivo* did not additionally damage the kidney. AlloTx experiments demonstrated a significant delay in the onset of rejection in treated kidneys in the absence of any systemic immunosuppression (107).

NPs are also attractive due to their ability to release the specific agents gradually. It has been found that pharmacological agents encapsulated within NPs can be incorporated into the endothelium, thereafter slowly releasing drugs by hydrolysis (160). This slow-release is especially interesting in handling the host's immune response against allografts, as it only evolves within several weeks after implantation. The work by Tietjen et al. focused on the improvement of NPs targeting kidney graft endothelium during NMP. Anti-human CD31 antibodies conjugated NPs were used to facilitate the binding and internalization of NPs by endothelial cells by delivery to non-transplantable human kidney grafts *via* NMP for 8.5 h. CD31-conjugated NPs accumulation was 5 to 10-fold higher than for non-conjugated NPs with no extravascular accumulation being observed. However, this increase was much less pronounced than observed *in vitro* where CD31-NPs accumulation was 80-fold higher than for control-NPs. Interestingly, non-specific NPs accumulated within the interstitial microvessels, where RBC-enriched vascular plugs were found. The extent of non-specific binding accordingly correlated to worse kidney perfusion. Nevertheless, despite the limitation of specific targeting of NPs in poor-perfused kidneys, the feature of non-specific accumulation of NPs could serve as a diagnostic/prognostic tool indicating vascular obstruction (108). The same group recently pre-treated non-transplantable human kidneys with tissue plasminogen activator (tPA) and plasminogen *via* NMP aiming ameliorate cold storage-caused microvascular obstructions to be able to deliver intercellular adhesion molecule (ICAM)-2-NPs. They observed a 3-fold increased retention of ICAM-2-targeted NPs in the glomeruli as well as ~20-fold increase in the microvessels meaning that more effective delivery of NPs and graft modification could be achieved by application of thrombolytics as an essential first step during NMP (109).

### Thrombolytics, Fibrinolytics, Anticoagulants

Formation of microthrombi is one of the most common problems in DCD kidneys or grafts retrieved from donors with disseminated intravascular coagulation, which often accompanies head injuries or multiorgan failure. Microvascular thrombosis can lead to poor graft perfusion and subsequent DGF or even PNF. Therefore, the discard rate of such kidneys remains high. Moreover, interactions among endothelial damage, inflammation, and activation of the coagulation system are well-known resulting in microthrombi, subsequent microcirculatory failure, and the “no-reflow” phenomenon as usual manifestations of IRI and acute antibody-mediated rejection (161, 162). As conventional systemic anticoagulation after kidney graft implantation does not eradicate pre-existing thrombi, does not guarantee the prevention of thrombosis, and carries its risk of bleeding (163, 164), the idea of anticoagulants or thrombolytics introduction directly to the graft has gained interest (165, 166). Several attempts of local thrombolysis or kidney pre-treatment with anticoagulants using *ex-vivo* machine perfusion have been published. Nghiem et al., used 14 human kidneys with biopsy-proven 50% thrombosed glomeruli that underwent 12–16 h of HMP with 200 mg tPA and were subsequently implanted. Reduction in glomerular thrombosis from 50 to 23% was observed at the end of perfusion, and 10 out of 14 recipients had immediate graft function after implantation (110). In the later RCT, after HMP with tPA, microthrombi were eradicated in all but one DCD kidney. No significant difference in kidney function, recipient survival, and death-censored graft survival could be observed which could also be due to the small study population (111). Despite promising results, it should be considered that tPA activity is reduced at lower temperatures suggesting that HMP, used in both studies, might not be optimal (167). DiRito et al. recently revealed that microvascular plugging in the kidney graft is induced by prolonged cold storage, which promotes fibrinogen production in proximal tubular cells and accumulation within the tubular epithelium. Upon restoration of physiological temperatures during NMP or implantation, rapid fibrinogen secretion to urine and microvasculature as well as RBC aggregation causes microvascular obstructions, occurring within 15 min after normothermic temperature restoration impairing adequate graft perfusion. Interestingly, those cold-induced obstructions seem to be different from traditional microthrombi. The investigators delivered tPA with plasminogen at the beginning of 1 h NMP of non-transplantable human kidneys and managed to clear microvascular obstructions completely. It subsequentially resulted in more stable perfusion parameters, decreased levels of NGAL, IL-6, and ICAM-1, and increased urine production. On the other hand, differently from the above-mentioned studies, neither tPA nor plasminogen alone had such an effect (109).

Another kidney *ex-vivo* perfusion study used thrombalexin, an endothelial localizing, cell membrane binding synthetic thrombin inhibitor. Nineteen ischemically damaged porcine kidneys and 2 non-transplantable human kidneys underwent 1.5 h of HMP with thrombalexin and were subsequentially hemoperfused for 6 h at physiologic temperature to mimic reperfusion. Perfect thrombalexin binding and adherence to graft microvasculature were confirmed after HMP remaining stable during 6 h of reperfusion. In comparison to the control group, treated kidneys demonstrated superior blood flow, 44% larger D capillaries, 50% faster RBC velocity, 3.5 times improved capillary blood flows and perfusion indices in orthogonal polarization spectral imaging, as well as lower d-dimer levels, confirming the anticoagulant activity of thrombalexin. Accordingly, cortical lactate levels were lower in treated kidneys, showing reduced ischemic damage and giving evidence that HMP could be successfully used to deliver cytotopic anticoagulant to the graft thus improving macro-and microvascular perfusion (114).

Sedigh et al. used HMP to deliver Corline heparin conjugate (CHC), a macromolecular heparin, consisting of >20 heparin molecules, to porcine DBD kidney grafts. CHC not only inhibits coagulation, platelet adhesion, and complement activity but also, differently from conventional heparin, irreversibly binds to collagen structures and therefore may be able to restore damaged endothelial glycocalyx and also locally express functional heparin (112, 113). In their first study, the group confirmed the safety and feasibility of CHC pre-treatment during HMP. It successfully bound to vessel walls of the ischemic kidneys, did not cause excess histological damage, or changed perfusion parameters (112). The later work on 3 h *ex-vivo* reperfusion showed that pre-coating vessels with CHC during HMP reduces preservation injury and improves organ function at least in the acute period. Treated kidneys had higher OU, faster decline in creatinine levels, lower urine NGAL levels, and less tubular damage in histological specimens (113).

Although none of those studies investigated the fate of delivered anticoagulant agents in longer periods after reperfusion and the actual need for systemic anticoagulation therapy afterwards, cytotopic delivery, and coating graft with anticoagulants during machine perfusion seems to be promising and provides a field for further studies before implementing this strategy into clinical practice.

### Gases

Several investigators used *ex-vivo* machine perfusion to deliver various gases (other than pure oxygen or 95% oxygen and 5% carbon dioxide mix) for kidney graft treatment. Multiple animal models revealed that such gaseous molecules, like carbon monoxide (CO) or NO, have cytoprotective, anti-inflammatory, antiapoptotic, or vasoregulatory effects and may be beneficial in the reduction of IRI or immune responses in the Tx setting (168–170). However, despite the long history of experiments, systemic donor or recipient treatment with gases never set foot in clinical practice due to side effects, difficulties to deliver gases in a safe and controlled manner, and logistic issues, which all might be solved by *ex-vivo* organ-specific treatment.

Hosgood et al. after 10 min of WI and 16 h of SCS, applied 2 h of NMP with NO donor sodium nitroprusside (SNP) or CO-releasing molecule (CORM-3) infused during the first hour of perfusion to porcine kidneys. Despite the short-acting time of these agents, the treatment not only increased RBF at the time of preservation but also improved hemodynamic parameters during 3 h of *ex-vivo* reperfusion. However, in this study, the renoprotective effects of CORM-3 were more apparent than for SNP in terms of RBF, oxygen consumption, UO, and CrCl. Moreover, the histological evaluation showed an increase in ischemic structural changes, such as tubular dilatation, vacuolation, and the number of condensed tubular nuclei in SNP-treated kidneys (115). In the later work, Bhattacharjee et al. used the fourth generation of CO releasing molecules CORM-401, which is more potent and allows more controlled CO release than previously synthesized molecules (117, 171). It has been demonstrated that at 37°C, CORM-401 releases 15 times more CO than at 5°C during the same period, suggesting that NMP could be superior to HMP in case of gas delivery. Another important finding of the study was that 200 μM of CORM-401 in plasmaLyte solution, delivered *via* the renal artery, over 10 h results only in a minimal and non-toxic level of carboxyhemoglobin (COHb) hence considering it safe. CORM-401 was delivered to kidneys originating from a porcine DCD model in a pulsatile manner at 37°C for 20 min followed by immediate reperfusion with autologous blood. This treatment increased RBF and total OU during reperfusion, reduced graft injury (lower histological acute tubular necrosis score, less necrosis, intrarenal hemorrhage, and apoptosis, diminished KIM-1 and NGAL levels in urine), urinary protein levels, and increased CrCl to compare with control kidneys. Interestingly, this treatment had an anti-inflammatory effect by significant downregulation of toll-like receptor (TLR)-2, 4, and 6, as well as MyD88, NF-κB, and HMGB1 genes expression (117).

A very recent study investigated the third gasotransmitter's (H_2_S) donor AP39 use during SNMP (21°C) (90). Its beneficial cytoprotective effects have already been demonstrated by supplementing preservation solutions of SCS in murine kidney IRI and Tx models (172, 173). Juriasingani et al. stored porcine DCD kidneys for 24 h in UW supplemented with AP39 at 21°C. Surprisingly, this strategy preserved kidneys better than SCS in UW without additives, giving rationale that delivery of H_2_S at higher temperatures could be even more beneficial (91). Indeed, SNMP with AP39 increased UO both during preservation and 4 h of normothermic reperfusion with stressed autologous blood. It also lowered the extent of apoptosis after reperfusion. RNA sequencing detected 214 genes, differentially expressed in the treatment group compared to the SCS group, including downregulated pro-apoptotic, heat shock response genes including regulators of those pathways as well as increased proliferation and oxidative stress response genes. When compared to SNMP without additives, the treatment group differentially expressed 614 genes with reduced expression of genes associated with the hypoxia inducible factor (HIF)-1α-mediated hypoxia response pathway, pro-inflammatory, and cell death-attenuating genes. On the contrary, the genes mediating proliferation, oxidative stress response, transforming growth factor (TGF)-β pathway, and HIF-1α degradation were upregulated (90).

Among other gases, 70% of argon has also been investigated as potential gasotransmitter for ischemically damaged porcine kidneys during 1 h of NMP. However, subsequent 3 h of *ex-vivo* reperfusion showed no measurable beneficial effect in graft histology, functional parameters, or inflammatory markers (116).

Nevertheless, current evidence suggests that *ex-vivo* delivery of gases, especially using gas-releasing molecules, is likely feasible and safe. Development of machine perfusion strategies and increasing experience of S(NMP) perfusion may be especially beneficial in targeted kidney graft treatment with gas not only due to more efficient gas release from gas-donor molecules to compare with hypothermic conditions but also due to easier control of such treatment *via* perfusion parameters. The efficacy still needs to be determined in auto- or alloTx models and subsequent clinical trials.

### Other Pharmacological Agents

#### EPO

Several studies investigated erythropoietin's (EPO) role in cytoprotection and preconditioning of kidney graft in the course of *ex-vivo* machine perfusion. Endogenous EPO is mainly produced by kidney cortical fibroblasts and not only participates in erythropoiesis but also acts locally through autocrine and paracrine axis *via* receptors in kidney tubular epithelium, endothelial cells, and mesangium. As a protective agent, it coordinates the response to injury of kidney cells by modulating pathways of apoptosis, necrosis, and inflammation (174). Nevertheless, to achieve a therapeutic effect, high doses of EPO are required. It vastly increases the risk of complications, such as hypertension and thrombosis, when administered systemically (17, 18). EPO contributes to tissue remodeling when added to NMP perfusate of ischemically damaged porcine kidneys. Two hours of NMP with EPO increased caspase-3 activity and the number of neutrophils, free cells, and cellular debris in tubular lumen but reduced the number of apoptotic cells and macrophages in tubulointerstitial areas compared to NMP alone. Reduced activity of pro-inflammatory cytokine IL-1β was also observed. Moreover, tubular apoptosis, dilatation, and cytoplasmic vacuolization in the tubular epithelium were significantly diminished, and UO increased in EPO-treated kidneys. The augmentation of apoptotic cells in tubular lumen but not in interstitial areas shows that EPO likely alleviates the clearance of dead inflammatory material, which could be beneficial for tissue conditioning before implantation and confrontation with the host's immune system (118). In another porcine study, cyclic helix B peptide, derived from the 3-dimensional structure of EPO using the cyclization method, was investigated. Such a structure improves cytoprotection but avoids induction of erythropoiesis and its related negative effects. Therefore, such peptide would be potent for systemic use. In this work, *ex-vivo* machine with whole blood as perfusate was used to simulate reperfusion and cyclic helix B peptide effect during the kidney reperfusion phase. Similar to the previous work, such treatment significantly decreased the number of apoptotic cells in the tubular areas, but increased in the lumen, again confirming EPO contribution in apoptotic cells clearance. Downregulation of caspase-3 expression (both precursor and active subunits) and upregulation of heat shock protein (HSP)70 in the treatment group was observed. Furthermore, cyclic helix B peptide improved the hemodynamic parameters RBF and oxygen consumption and increased UO. Even though drug administration during reperfusion is outside of the subject of this review, the results of this study suggest that cyclic helix B peptide could be used as an alternative to conventional EPO during the preservation phase for improved organ conditioning and protection (119).

#### Metformin

Huijink et al. investigated the potential beneficial effect of metformin on kidney graft injury by the donor pre-treating and/or adding it to NMP solution in rats and pigs experiments (120). Metformin is not only known as an antihyperglycemic agent but also has some pleiotropic effects, due to its ability to inhibit the complex 1 of the mitochondrial respiratory chain, coordinate cellular energy state, inhibit apoptosis, and regulate endothelial function *via* NO production (175, 176). Organo-protective action of metformin has also been demonstrated in an ischemia-reperfusion setting (177, 178). Nevertheless, the results of the Huijink et al. study were inconclusive as the beneficial effect on kidney integrity was observed mainly in rodent experiments when metformin was used for preconditioning (donor treatment). However, perfusate supplementation during NMP was not as effective. Nevertheless, downregulation of eosinophil-derived neurotoxin (EDN)-1, eNOS, vascular cell adhesion molecule (VCAM)-1, IL-6 genes in rat kidneys and upregulation of HSP-70 in porcine kidneys was observed. Moreover, metformin-perfused kidneys revealed lower kidney injury in histological specimens, encouraging further investigation of metformin-treatment using an *ex-vivo* perfusion platform (120).

#### Doxycycline

Another kidney graft pharmacological treatment strategy is inhibition of matrix metalloproteinases, which play a role in the pathogenesis of ischemia-reperfusion (179), as well as acute and chronic immune injury (180, 181). Activation of MMPs leads to acute tubular injury, necrosis, apoptosis, tubular atrophy, fibrosis, and damage of the basal membrane in the kidney (179, 182). Moser et al. observed increased levels of MMP-9 and MMP-2 during HMP both in human and rat kidneys, along with elevated levels of total protein, NGAL, and LDH in perfusate, indicating preservation injury. Twenty-two hours of rat kidney HMP resulted not only in higher levels of MMPs and kidney injury markers but also in structural impairment of mitochondrial integrity compare to kidneys perfused for only 5 h. Upon supplementing HMP perfusate with doxycycline, which is not only an antibiotic but also a clinically approved MMP inhibitor, 22 h HMP levels of MMP-2 and MMP-9, LDH, cytochrome c oxidase, NGAL, and total protein dropped to the levels of 5 h perfusion without treatment. Moreover, the doxycycline effect on mitochondrial membrane protection was demonstrated by electronic microscopy (104). Extensive proteomic analysis showed a significant increase in 8 proteins in doxycycline perfused rat kidneys, including glycolysis enzymes triosephosphate isomerase (TPI), phosphoglycerate kinase 1 (PK-1), phosphoglycerate mutase (PGM), urea cycle enzyme aminoacyclase-1A, NO synthesis regulator N(G),N(G), dimethylarginine dimethylaminohydrolase, as well as, other enzymes, such as dihydropteridine reductase-2, pyridine nucleotide-disulfide oxidoreductase and phosphotriesterase-related protein. Interestingly, TPI, PK-1, and N(G),N(G), dimethylarginine dimethylaminohydrolase levels were reduced by HMP, while treatment with doxycycline allowed correction of this reduction, again proving mitochondrial preservation (121).

#### SUL Compounds

Another class of novel agents that protect cells from hypothermia-associated damage by preserving mitochondrial structure and function and reactive oxygen species (ROS) scavenging are 6-chromanol derivates (SUL compounds) (183, 184). Nakladal et al. added SUL-121 and its enantiomers SUL-150 and SUL-151 into the NMP solution of porcine kidneys after 24 h of SCS. An apparent and immediate increase in RBF and decrease in intrarenal pressure, mainly through SUL-150 enantiomer, was observed. *In vitro* experiments with isolated intraarterial arteries using various agonists showed that the beneficial vascular effect of SUL-121/SUL-150 is mediated by specific competitive inhibition of α1-adrenoreceptors on the vascular smooth muscle. This study did not investigate SUL-121 impact on ROS production and mitochondrial function when it is administered specifically during machine perfusion. Therefore, although *ex-vivo* kidney graft treatment with 6-chromanol derivates after prolonged cold ischemia seems feasible and promising, further investigation is necessary (122).

#### Propofol

Besides the anesthetic properties, the well-known agent propofol has common structural elements like α-tocopherol and acts as antioxidant by preventing lipid peroxidation in cell membranes (185, 186). Snoeijs et al. investigated renoprotective features of propofol by delivering it to ischemically damaged porcine kidney grafts *via* 22 h HMP. To make it water-soluble, they prepared a cyclodextrin inclusion complex. The treatment prevented lipid peroxidation, reduced the increase in renovascular resistance at reperfusion after autologous kidney implantation. Moreover, treatment with propofol slightly improved kidney function in the early period after Tx. However, leucocyte infiltration in kidneys was not diminished by propofol treatment. The main advantage of HMP delivery was that it allowed high tissue concentrations of the agent without any adverse effects after graft implantation. Propofol concentrations were undetectable in recipients' plasma in the early reperfusion periods (123).

### Hemadsorbtion

It has been recently found that, despite leukocytes- and complement-free perfusate, kidney tubular epithelial cells and circulating cells locally produce cytokines and chemokines, hence up-regulating inflammatory pathways within the graft already during *ex-vivo* machine perfusion (59, 65). As it is well-known that pro-inflammatory mediators aggravate the severity of IRI, the removal of cytokines and chemokines from the kidney using machine perfusion seems to be a logical strategy of organ preparation before implantation. This question was analyzed in two recent experimental studies (64, 65). Cytosorb hemadsorbtion filter, which is currently widely used in intensive care to treat severe inflammatory states, such as systemic inflammatory response syndrome and sepsis (187, 188), was connected to the perfusion machine and used to remove cytokines from perfusate during kidney NMP (64, 65). In the first study on porcine kidneys, a significant increase of IL-1β, IL-1α, IL-1RA, TNFα, IL-10 was observed after 6 h of NMP in control kidneys, whereas levels of these cytokines remained low in the hemadsorbtion group. Decreased levels of IL-6, IL-8, c reactive protein (CRP), and thromboxane B2 were also observed in treated kidneys. No effect on UO, CrCl, and FENa was observed, but kidney injury marker NGAL levels in urine were significantly lower in the Cytosorb group (probably due to direct filtering of the NGAL molecule). Interestingly, even though overall blood flow throughout 6 h of NMP was increased in the treatment group a rapid decrease in RBF was observed at 30 min of perfusion, following a decline of vasodilators prostaglandin E (PGE) and prostacyclin. Cytosorb advantage is the ability to filter a wide range of mediators according to their size (10–50 kDa). However, it is not specific and also removes anti-inflammatory agents, such as IL-10 or IL-1RA, and vasodilators PGE and prostacyclin. This problem could be partly solved by adding vasodilators to the perfusate when hemadsorbtion is used (64). Ferdinand et al., used the same technique to perfuse non-transplantable human kidney pairs, focused on changes in graft gene expression after cytokine removal. They showed that NMP has a double effect on gene regulation. It promotes oxidative phosphorylation pathway genes, which are crucial for energy generation. However, at the same time, several pro-inflammatory and immune pathway genes are induced. Using the samples from the clinical study of kidney NMP, the investigators found that this “gene signature” is associated with prolonged DGF after implantation. In non-transplantable human kidneys, NMP with Cytosorb hemadsorbtion not only diminished levels of cytokines but also significantly reduced inflammatory gene expression and up-regulated genes of the oxidative phosphorylation pathway. These surprising results suggest that locally produced and during NMP recirculating cytokines likely play a role in kidney gene regulation and its shift toward a pro-inflammatory state, culminating in aggravated graft injury after implantation. Hemadsorption during *ex-vivo* kidney perfusion seems to be a promising approach to cease this inflammation exacerbating loop however, more experimental studies followed by kidney implantation and clinical studies are necessary to strengthen this hypothesis (65).

## Challenges and Considerations

In the last decade, significant progress has been made in the kidney preservation field. Many *ex-vivo* machine perfusion therapeutic strategies have been developed and proved feasible in multiple experimental and a few clinical studies. Nevertheless, the kidney machine perfusion therapeutics is still in its infancy therefore, several challenges remain and need to be considered.

Currently, among other *ex-vivo* kidney perfusion strategies, only HMP is fully established and routinely used in clinical practice. Therefore, it could be the fastest and easiest way to implement kidney machine perfusion therapeutics into clinical practice. Indeed, several studies demonstrated a successful delivery of MSC and EV (97), siRNA (104), fibrinolytics and anticoagulants (110–114), doxycycline (104, 121), and propofol (123). However, the pharmacokinetics and pharmacodynamics of different agents at 4°C is not well-known (105). The possibility that low temperature might reduce the effectiveness of the therapy (167) or even enhance the accumulation of the agent, which can be damaging after reperfusion, should be considered. Moreover, the kidney outcomes after HMP still depend on CIT (30, 42). Therefore, it could be used only for several hours of therapy, which might not be sufficient for tissue regeneration strategies.

NMP, which ideally provides a physiological environment, is a more attractive platform for kidney *ex-vivo* therapeutics. Not surprisingly, most of the experimental studies used physiological temperature for kidney graft therapy. Long-term warm perfusion is crucial to enhance kidney tissue repair and regeneration (62, 79). SNMP was also found to be effective, especially for gas delivery (90). However, none of these strategies have been adopted in clinical practice yet. The results of the first clinical trial of short-term end-ischemic NMP should answer some questions and probably even open the window of opportunities for kidney perfusion therapeutics (69).

Nevertheless, the optimal protocol, especially for long-term perfusion, still needs to be established including, optimal timing, perfusion settings, oxygen partial pressure or perfusate composition. Moreover, the discussion regarding kidney evaluation parameters during perfusion, which is crucial to observe the graft state during therapy, is still ongoing The rationale for the use of perfusion readings, such as pressure, flow and resistance, is based on the kidney structure, which is rich in capillary network with filtration function. The release of vasoconstrictors from capillaries following the ischemic and inflammatory insults, determines accumulation of erythrocytes and microthrombosis, leading to a diminished flow and increased resistance in the graft. The hypoxia directly activates single-layer endothelium cells, favoring a pro-coagulant and pro-inflammatory phenotype of the kidney vasculature, with consequent disruption of the blood flow, increased leukocyte infiltration, and a further decline in kidney function. On the basis of kidney structure and physiology, perfusion readings are considered as a valuable tool to assess kidney viability before implantation. On the other hand, the relative predictive value of perfusion parameters is low and they cannot be considered as the only criteria to determine, whether to transplant the kidney. In general, there is likely no universal perfusate marker or universal perfusion parameter. However, the best combination of different parameters, sensitive and specific kidney quality score, is an objective of the current research (22). The optimal timing and duration of the different treatments during kidney perfusion also requires further investigation. Due to the short action or life-span of many agents, it might be necessary to continue some therapies systemically after implantation.

The main limitation of the aforementioned studies is that the results mostly reveal the expression of different molecular markers, and thorough investigation of the treatment impact on kidney function after implantation, clinical alloimmune response and transplant outcomes is lacking. Most of the experiments only evaluated the preservation stage or used short *ex-vivo* reperfusion models, which might not reflect the real conditions after implantation. Therefore, more animal models of kidney implantation and longer follow-up periods are necessary before kidney perfusion therapeutics clinical studies. Lastly, implementation of novel gene and cell therapies might be challenging due to ethical regulation, logistics (e.g., MSC retrieval and growth), and high cost. On the other hand, the application of those therapies *ex-vivo via* the machine perfusion platform is more ethically acceptable and less risky than systemic recipient or donor treatment. Therefore, machine perfusion might facilitate the adoption of novel techniques and therapies into clinical practice.

## Conclusions

The rapid development of kidney *ex-vivo* machine perfusion techniques and strategies started a new era in kidney Tx. Many (pre-) clinical studies on *ex-vivo* kidney perfusion using various modalities have already demonstrated its safety and feasibility. Further, IRI and graft immunogenicity may be reduced, and both regeneration and repair may be promoted. Kidney perfusion therapeutics offers an opportunity to overcome the challenges faced with ECD or DCD kidneys, increase the pool of transplantable organs and improve outcomes after implantation. Nevertheless, further extensive research is necessary to transfer these techniques from the experimental to clinical stage.

To date criteria for kidney machine perfusion to improve graft survival/function are still pending. Clinical trials are warranted to define organs that need *ex-vivo* machine perfusion before transplantation for optimal graft function. These data would allow a cost-effective use of machine perfusion.

## Author Contributions

PSt was responsible for the study concept design and critical revision of the drafted manuscript. RZ, PM, PSc, BL, and KS were responsible for the literature review, interpretation, and drafting of the manuscript. All authors have read and agreed to the published version of the manuscript.

## Conflict of Interest

The authors declare that the research was conducted in the absence of any commercial or financial relationships that could be construed as a potential conflict of interest.

## Publisher's Note

All claims expressed in this article are solely those of the authors and do not necessarily represent those of their affiliated organizations, or those of the publisher, the editors and the reviewers. Any product that may be evaluated in this article, or claim that may be made by its manufacturer, is not guaranteed or endorsed by the publisher.
